# Supplementary material for: Myr-Arf1 conformational flexibility at the membrane surface sheds light on the interactions with ArfGAP ASAP1
Source: Nat Commun. 2023 Nov 21;14:7570. doi: 10.1038/s41467-023-43008-5 (PMC10663523; doi:10.1038/s41467-023-43008-5)

A

```
2         11          21          31          41          51
|          |           |           |           |           |
GNIFANLFKG  LFGKKEMRIL  MVGLDAAGKT  TILYKLKLGE  IVTTIPTIGF

52        61          71          81          91          101
|          |           |           |           |           |
NVETVEYKNI  SFTVWDVGGQ  DKIRPLWRHY  FQNTQGLIFV  VDSNDRERVN

102       111         121         131         141         151
|          |           |           |           |           |
EAREELMRML  AEDELRDAVL  LVFANKQDLP  NAMNAAEITD  KLGLHSLRHR

152       161         171         181         191
|          |           |           |           |
NWYIQATCAT  SGDGLYEGLD  WLSNQLRNQK  GSGSHHHHHH
```

B

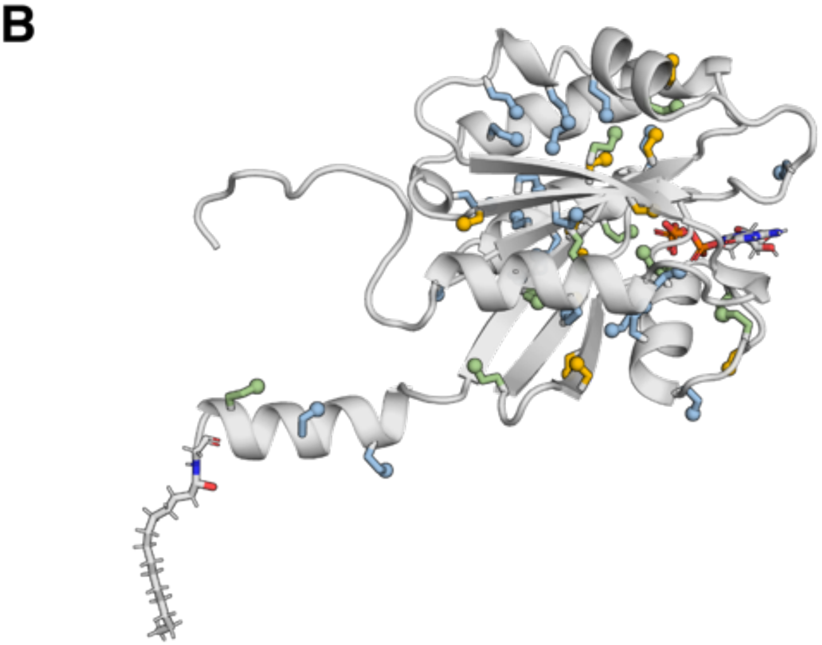

Supplementary Figure1. myr-Arf1 sequence and distribution of methyl probes used as reporters for NMR experiments. A) Sequence of human myr-Arf1, including the C-terminal hexa-histidine tag. The myristoyl chain is post-translationally attached to Gly-2 during protein expression. Isoleucine, leucine, and valine residues are colored green, blue, and yellow, respectively. B) Ribbon representation of the homology model of human Arf1 generated from yeast Arf1 (PDB:2KSQ [https://doi.org/10.2210/pdb2KSQ/pdb]) by MODELLER[1]. The ball-and-stick structures are colored using the same scheme as in the panel A. Residues 2-13 form the N-terminal helix, Residues 17-181 form the G domain. For leucine and valine residues, only the *Pro-S* methyl carbons are shown.

A

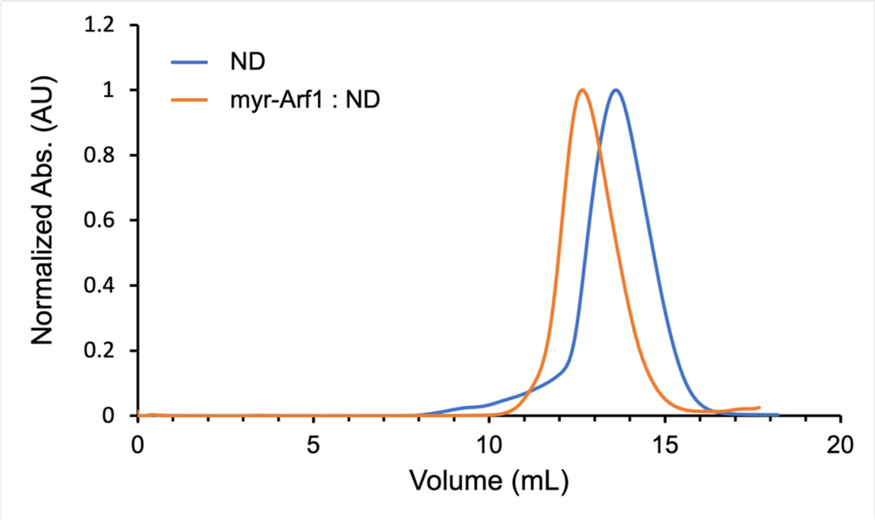

B

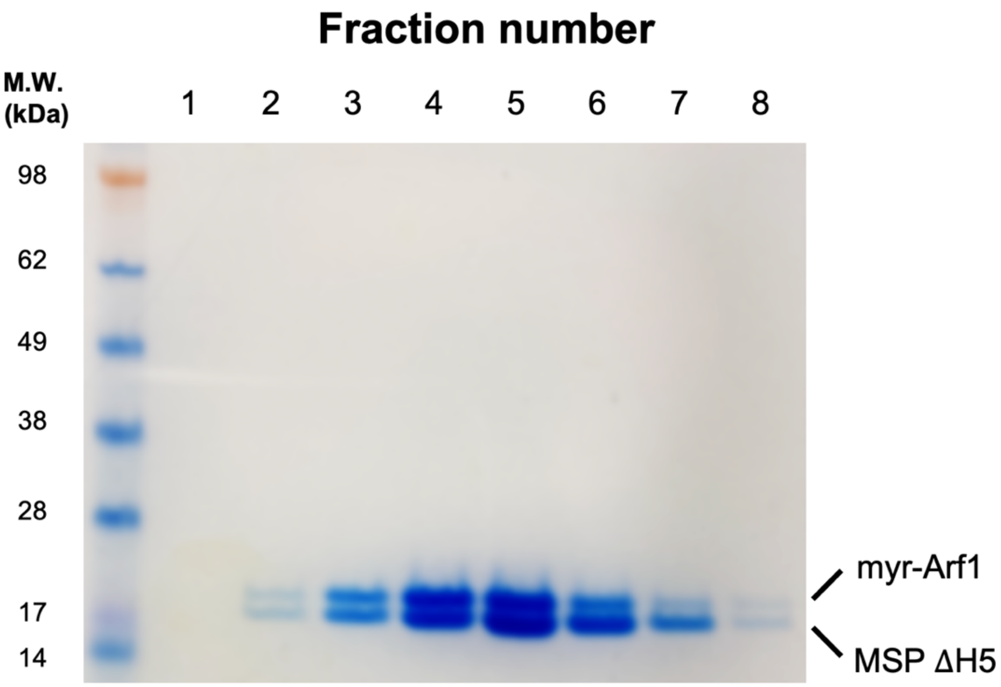

Supplementary Figure 2. Preparation of myr-Arf1:ND complexes. A) Comparison of size-exclusion chromatography (SEC) profiles of MSP ΔH5 nanodisc with (blue) and without (orange) bound- myr-Arf1•GTP. B). SDS-PAGE gel of SEC fractions (0.75 mL/fraction taken in the 10-15 mL elution range of panel A, beginning with fraction 1 at 10.0-10.75 mL) selected from the peak corresponding to the myr-Arf1:nanodisc complex. Fraction numbers 2-5 were selected based on the equal ratio between myr-Arf1 and MSP Coomassie Blue stained gel band intensities, suggesting that each nanodisc harbors 2 myr-Arf1•GTP, as previously observed[2]. Source data are provided as a Source Data File.

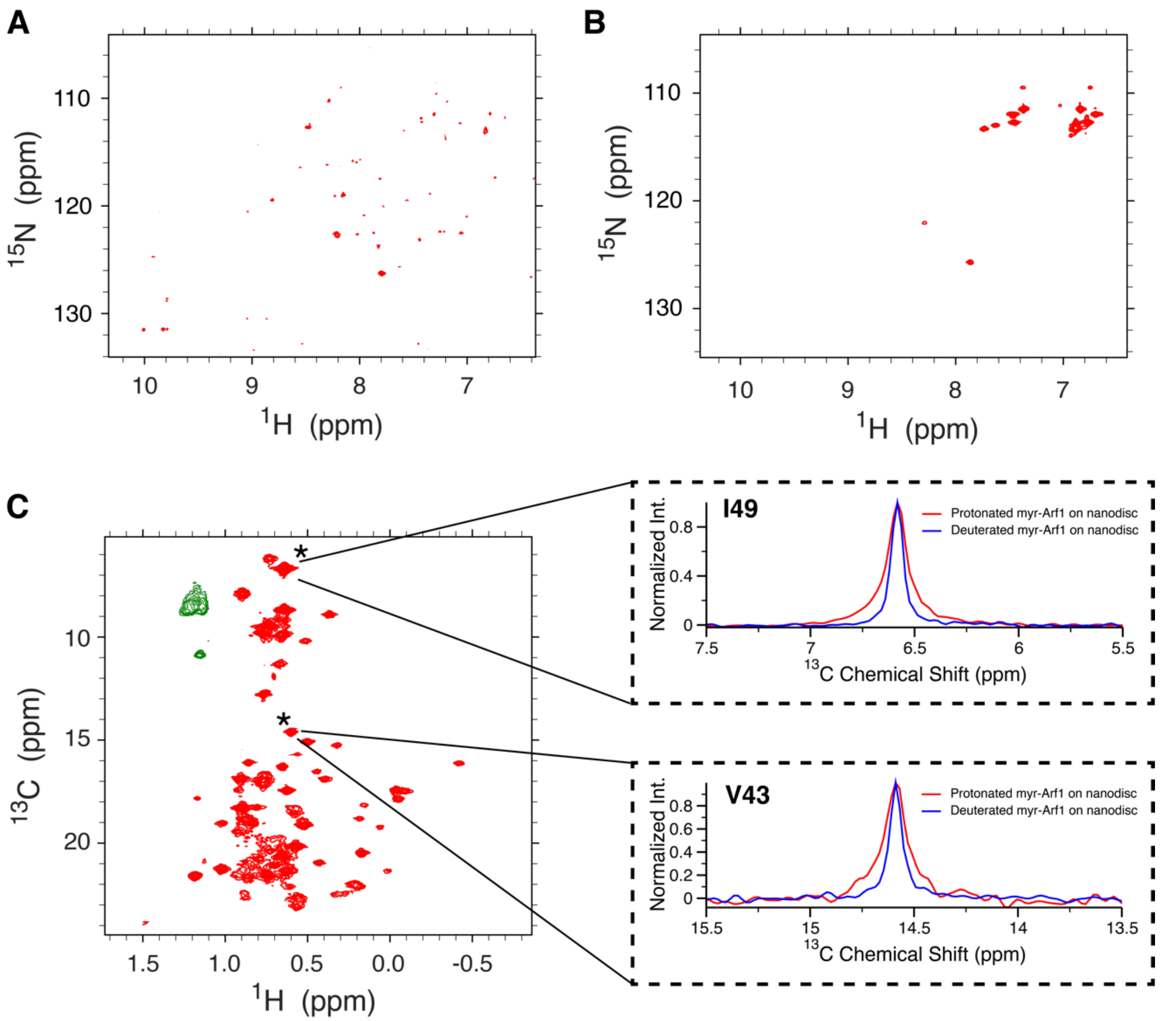

Supplementary Figure 3. Influence of deuteration on the backbone amide and ILV side chain methyl spectra of myr-Arf1:nanodisc complexes. In panels A-C are ^{1}H-^{15}N TROSY, ^{1}H-^{15}N HSQC, and ^{1}H-^{13}C HMQC spectra (25 ºC) of 200 μM protonated myr-Arf1-GTP on the nanodisc (DMPC:PI(4,5)P_2 = 95:5), respectively. Compare to Figure 1C-E for deuterated myr-Arf1 complexes. The contour level was adjusted to show all observable peaks without noise. Two insets in panel C illustrate 1D slices (along the ^{1}H axis) at the ^{13}C position of the I49 and V43 resonances: protonated, $^{13}CH_3$-labeled myr-Arf1 in red; deuterated, $^{13}CH_3$-labeled myr-Arf1 in blue. The same processing parameters were used for both the protonated and deuterated HMQC spectra. The linewidth differs by approximately two-fold, and the sensitivity difference is also approximately two-fold. Source data are provided as a Source Data File.

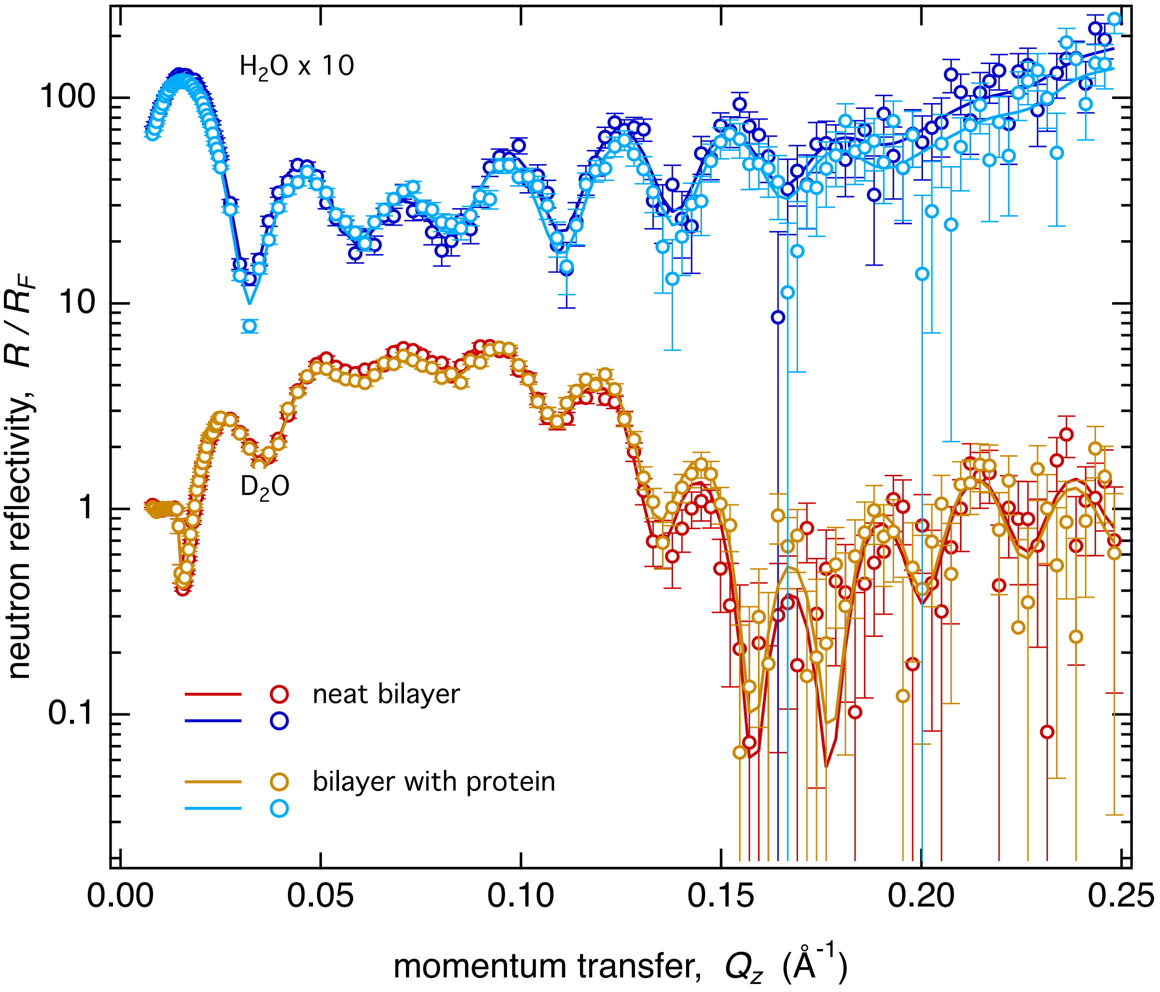

Supplementary Figure 4. Neutron reflectivity curves for the 85:15 POPC:POPS stBLM before and after adding myr-Arf1. Each condition has been measured twice, subsequently immersing the stBLM in D_2O- or H_2O-based buffer**.** Error bars represent 68% confidence limits. The statistical analyses of the fit of the reflectivity were carried out with the reduced χ2 method using composition-space modeling and the Monte-Carlo Markov chain global optimizer DREAM as implemented in the Refl1D software package[3]. Source data are provided as a Source Data File.

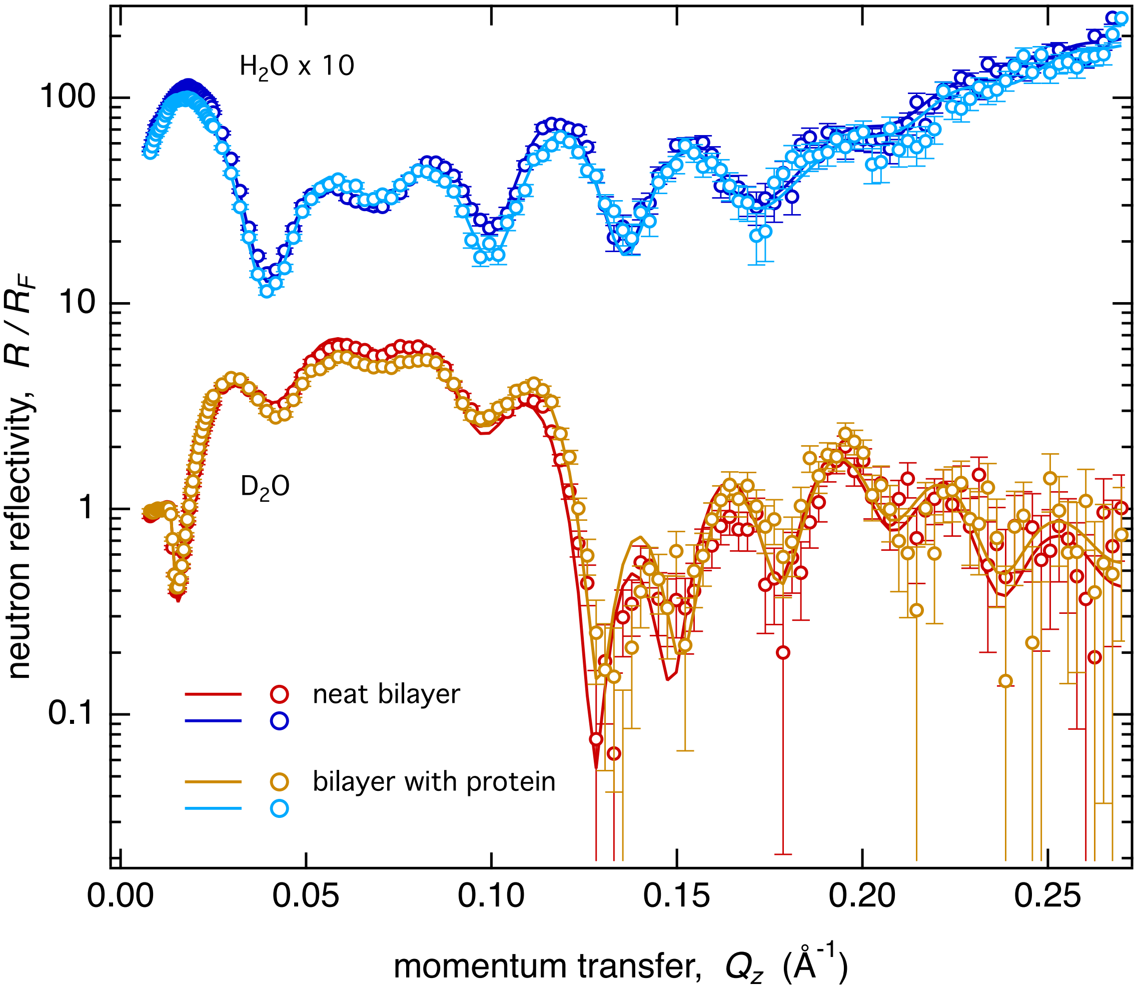

Supplementary Figure 5. Neutron reflectivity curves for the 95:5 POPC : PI(4,5)P_2 stBLM before and after adding myr-Arf1. Each condition has been measured twice, subsequently immersing the stBLM in D_2O- or H_2O-based buffer. Error bars represent 68% confidence limits. The statistical analyses of the fit of the reflectivity were carried out with the reduced χ2 method using composition-space modeling and the Monte-Carlo Markov chain global optimizer DREAM as implemented in the Refl1D software package[3]. Source data are provided as a Source Data File.

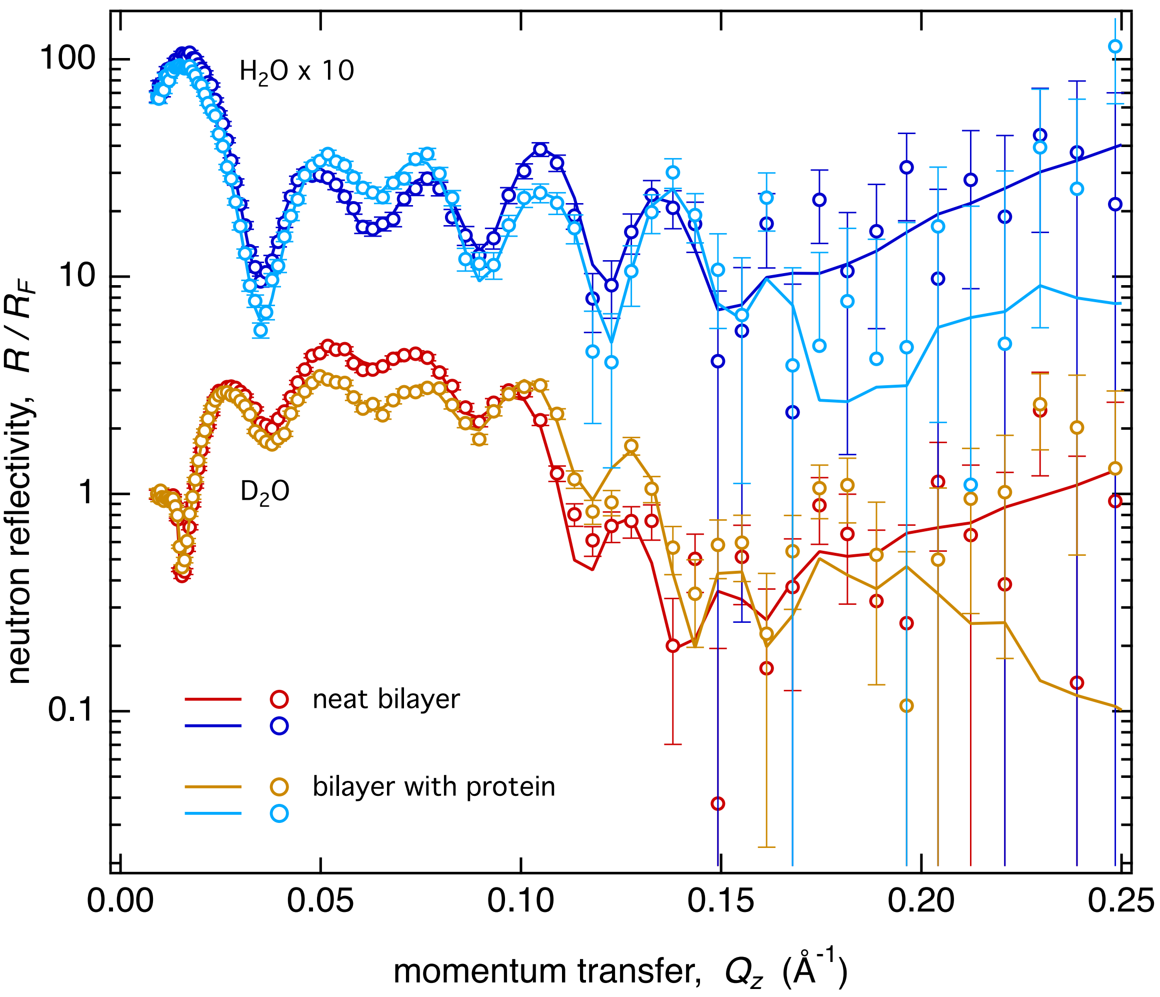

Supplementary Figure 6. Neutron reflectivity curves for the 87:10:3 POPC:POPS:PI(4,5)P_2 stBLM before and after adding myr-Arf1. Each condition has been measured twice, subsequently immersing the stBLM in D_2O- or H_2O-based buffer. Error bars represent 68% confidence limits. The statistical analyses of the fit of the reflectivity were carried out with the reduced χ2 method using composition-space modeling and the Monte-Carlo Markov chain global optimizer DREAM as implemented in the Refl1D software package[3]. Source data are provided as a Source Data File.

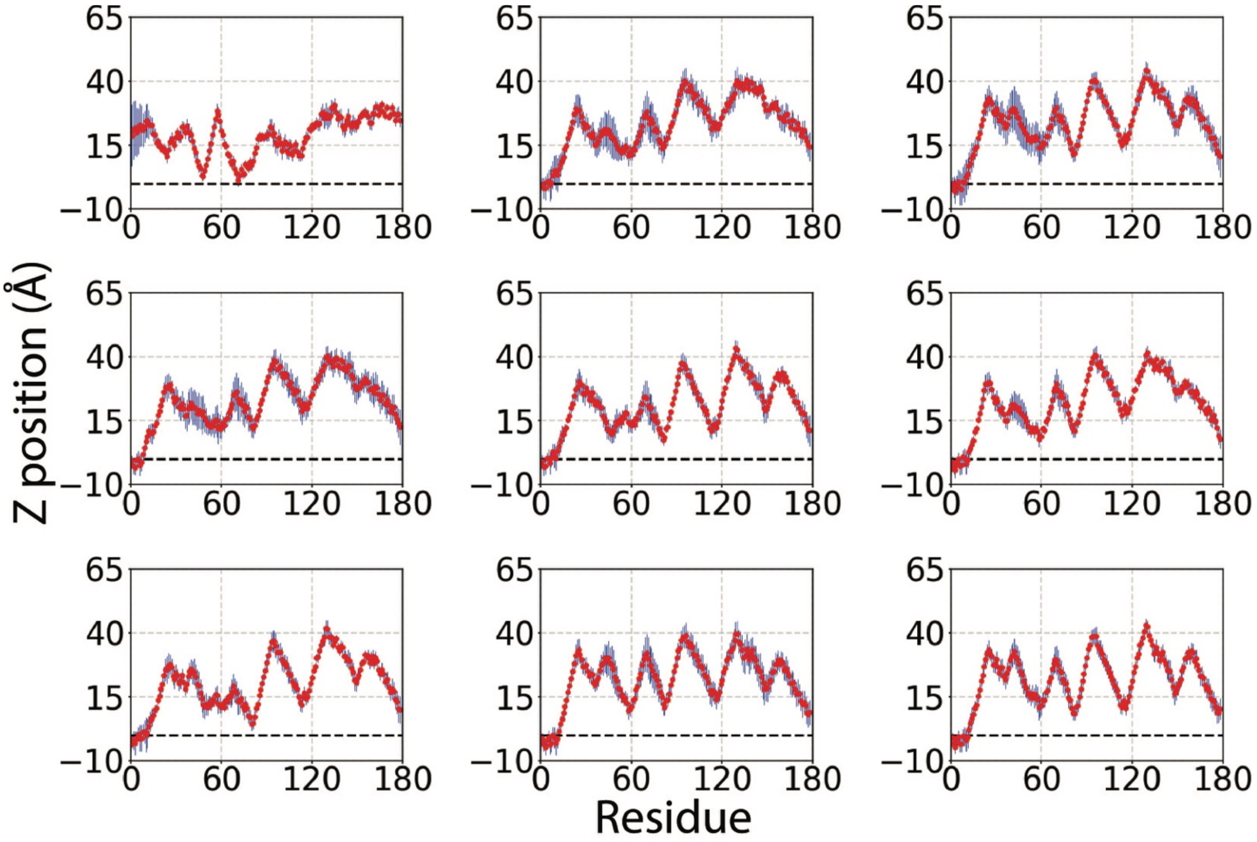

Supplementary Figure 7. Ensemble-averages C_{α} profiles of Arf1 residue insertion into the membrane calculated during the last 50 ns of the 9 replicas of the HMMM membrane-binding simulations in the presence of pure POPC lipid bilayers. In 8 out of the 9 simulations, the N-terminal helix was observed to penetrate into the membrane below the phosphorus plane (dashed lines) of the lipid bilayer. Standard deviations are shown as blue bars. Source data are provided as a Source Data File.

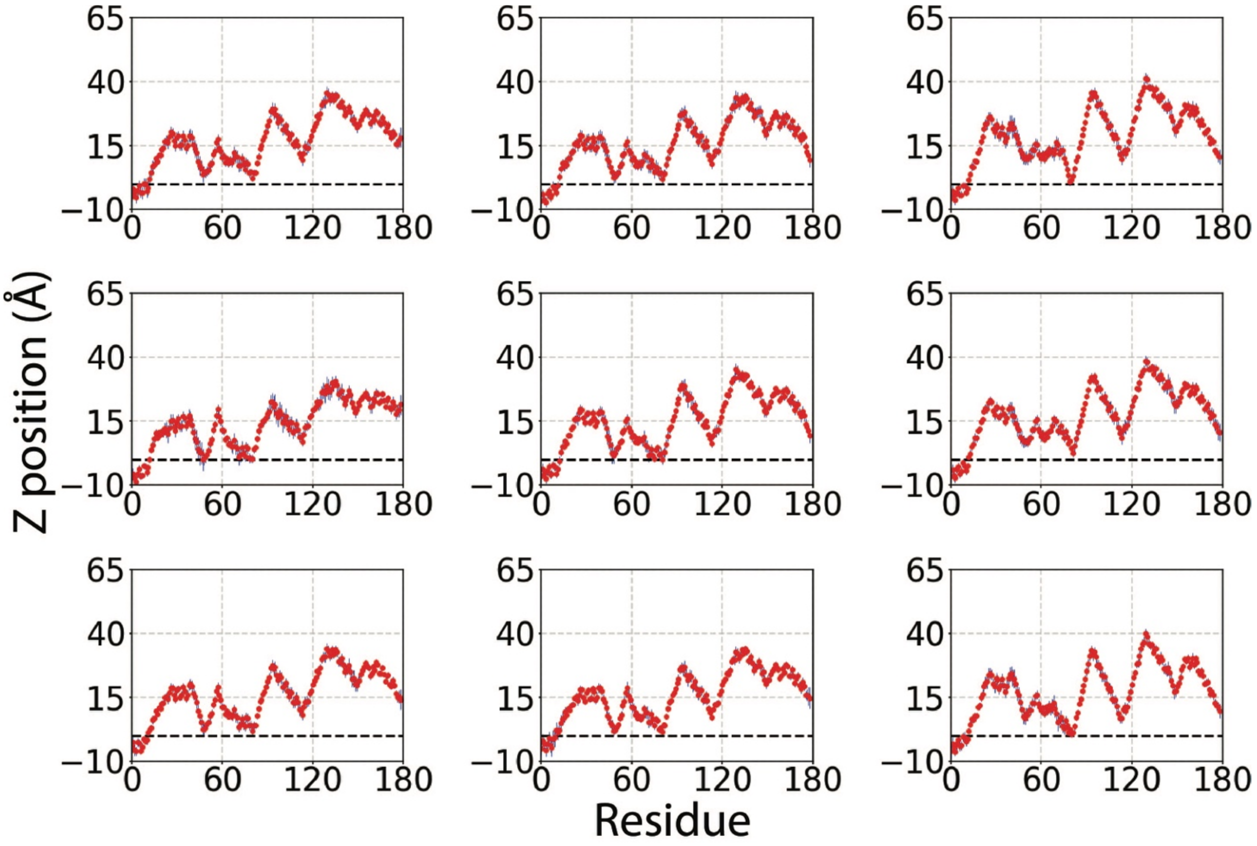

Supplementary Figure 8. Ensemble-averages C_α profiles of Arf1 residue insertion into the membrane calculated during the last 50 ns of the 9 replicas of the HMMM membrane-binding simulations in the presence of POPC:POPS:PI(4,5)P_2 (8:1.5:0.5) lipid bilayers. In all the simulations, the N-terminal helix was observed to penetrate into the membrane below the phosphorus plane (dashed lines) of the lipid bilayer. Standard deviations are shown as blue bars. Source data are provided as a Source Data File.

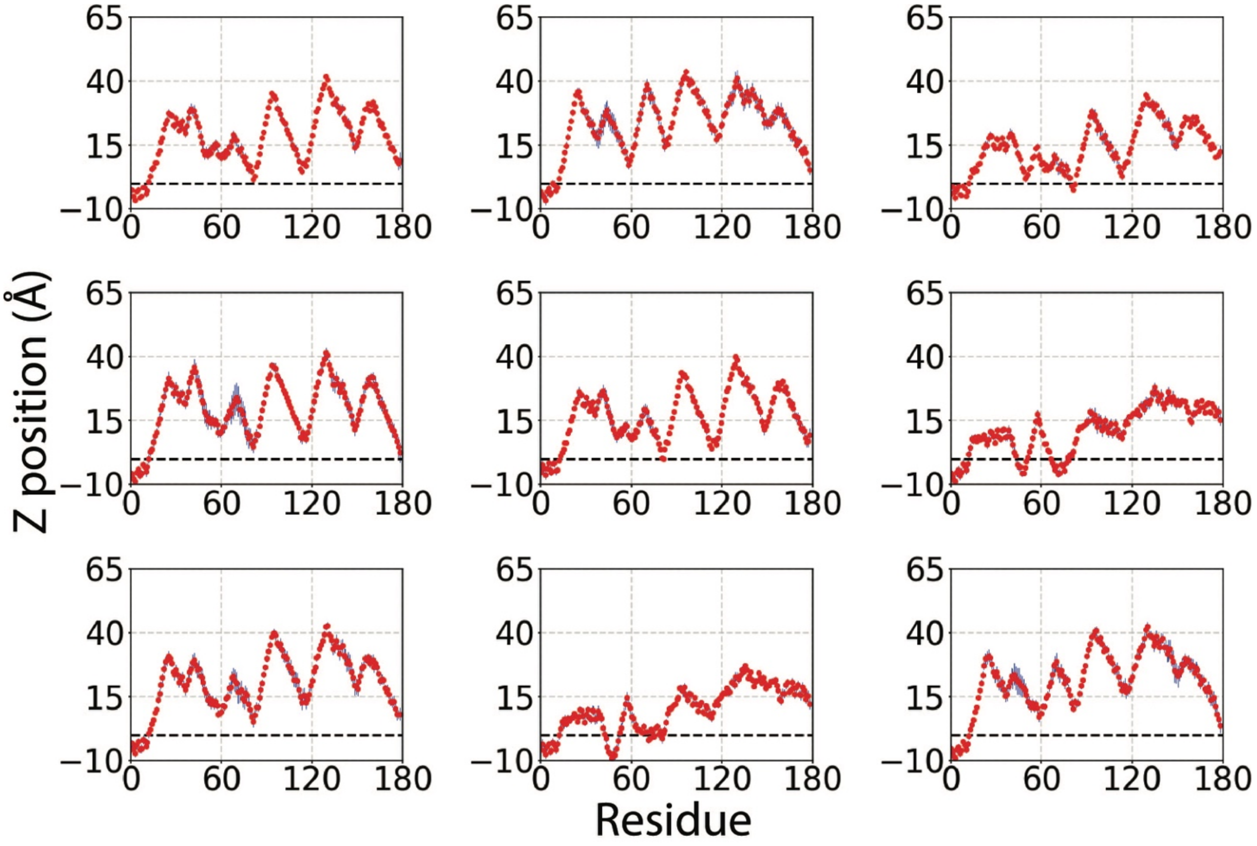

Supplementary Figure 9. Ensemble-averages C_{α} profiles of Arf1 residue insertion into the membrane calculated during the last 50 ns of the 9 replicas of the HMMM membrane-binding simulations in the presence of pure POPC:PI(4,5)P_2 (9.5:0.5) lipid bilayers. In all the simulation replicas, the N-terminal helix was observed to penetrate into the membrane below the phosphorus plane (dashed lines) of the lipid bilayer. Standard deviations are shown as blue bars. Source data are provided as a Source Data File.

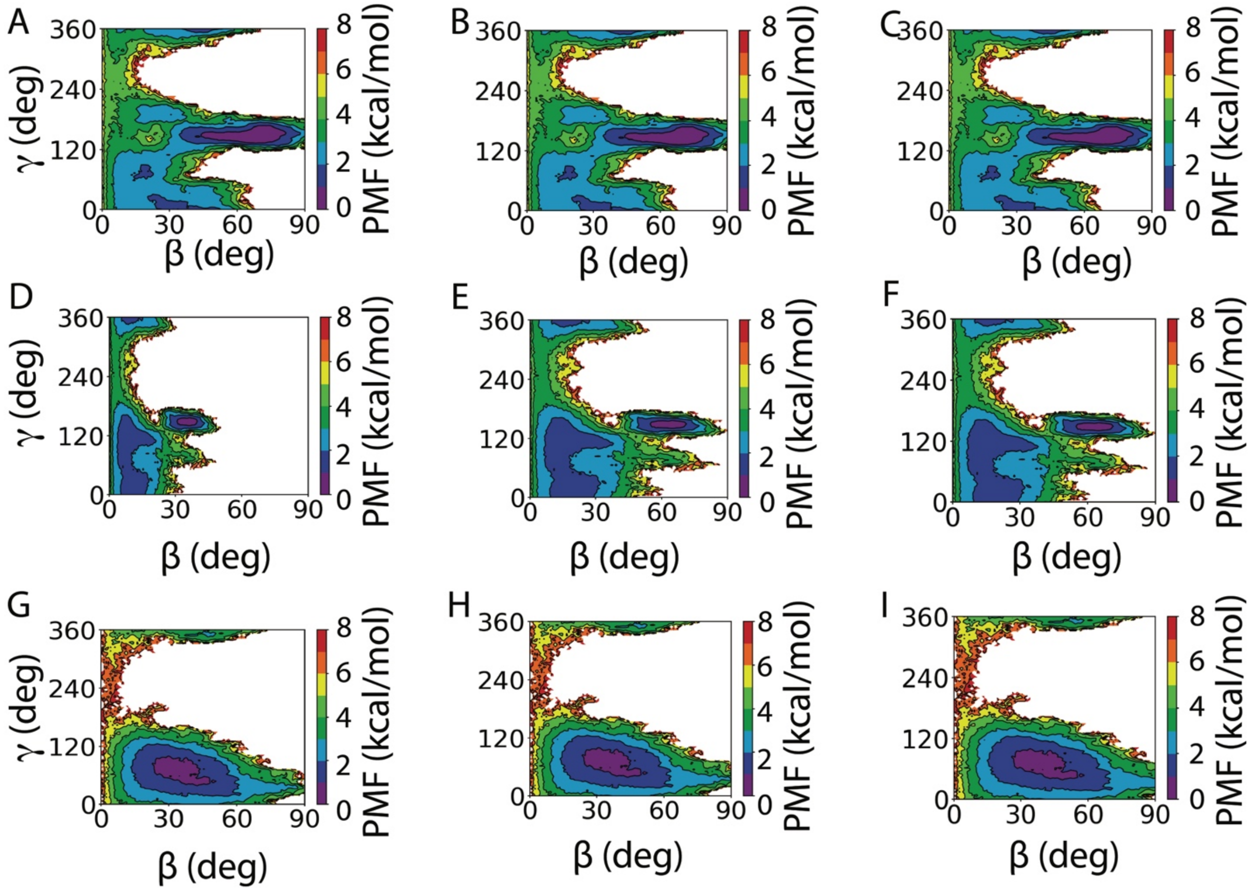

Supplementary Figure 10. Convergence of 2D PMF in three different lipid environment was assessed by recalculating them considering only a subset of simulation data. 2D PMF plots corresponding to PC-PS-PIP2 containing lipid bilayer by considering (A) 50%, (B) 75% and (C) 90% of the simulation data. 2D PMF plots corresponding to PC-PIP2 containing lipid bilayer by considering (D) 50%, (E) 75%, and (F) 90% of the simulation data. 2D PMF plots corresponding to pure PC containing lipid bilayer (G) 50%, (H) 75%, and (I) 90% of the simulation data. Source data are provided as a Source Data File.

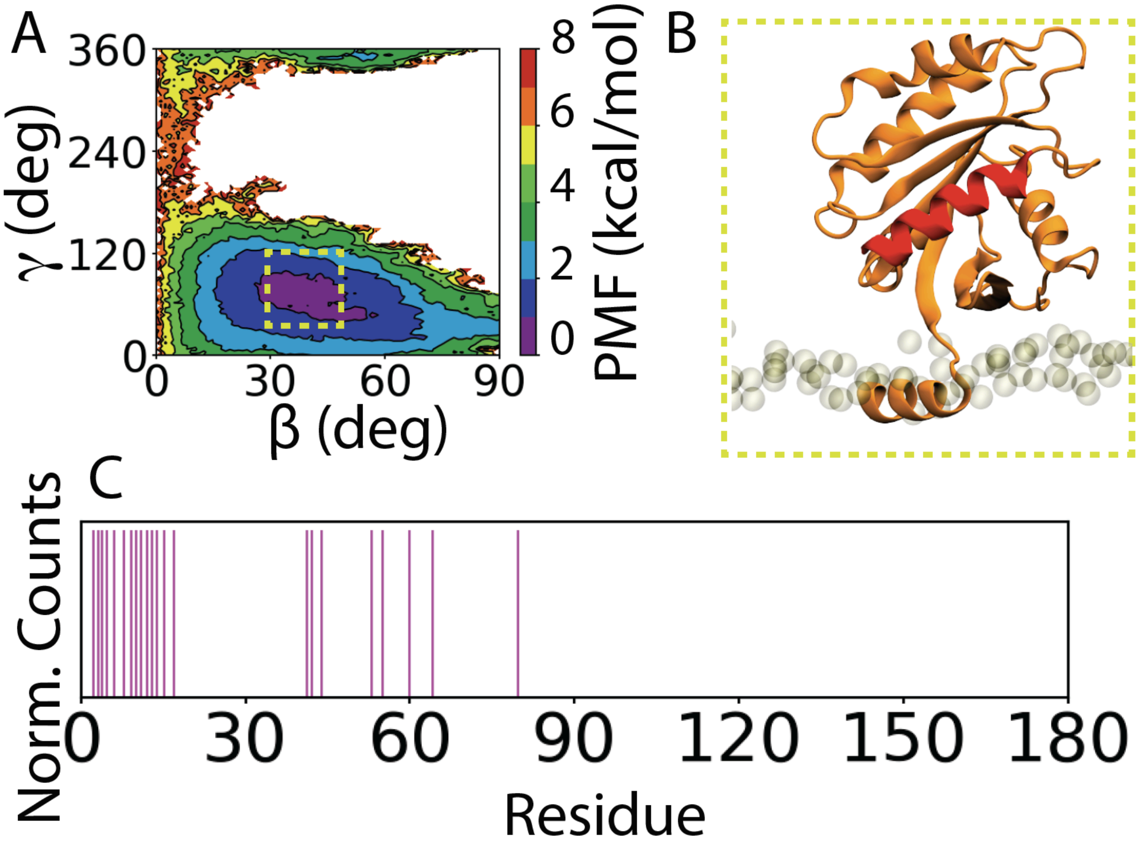

Supplementary Figure 11. Differential orientational dynamics of Arf1 in the presence of pure POPC lipids. (A) We captured a single dominant population of Arf1 in the presence of pure POPC membranes (highlighted in the yellow box). In the presence of anionic membranes, the energy well was relatively broad suggesting a relatively more dynamic nature of the protein. (B) Representative snapshot of the membrane-bound protein corresponding to the energy well shown in A. (C) Specific lipid-protein interactions in the captured conformation of the protein. Source data are provided as a Source Data File.

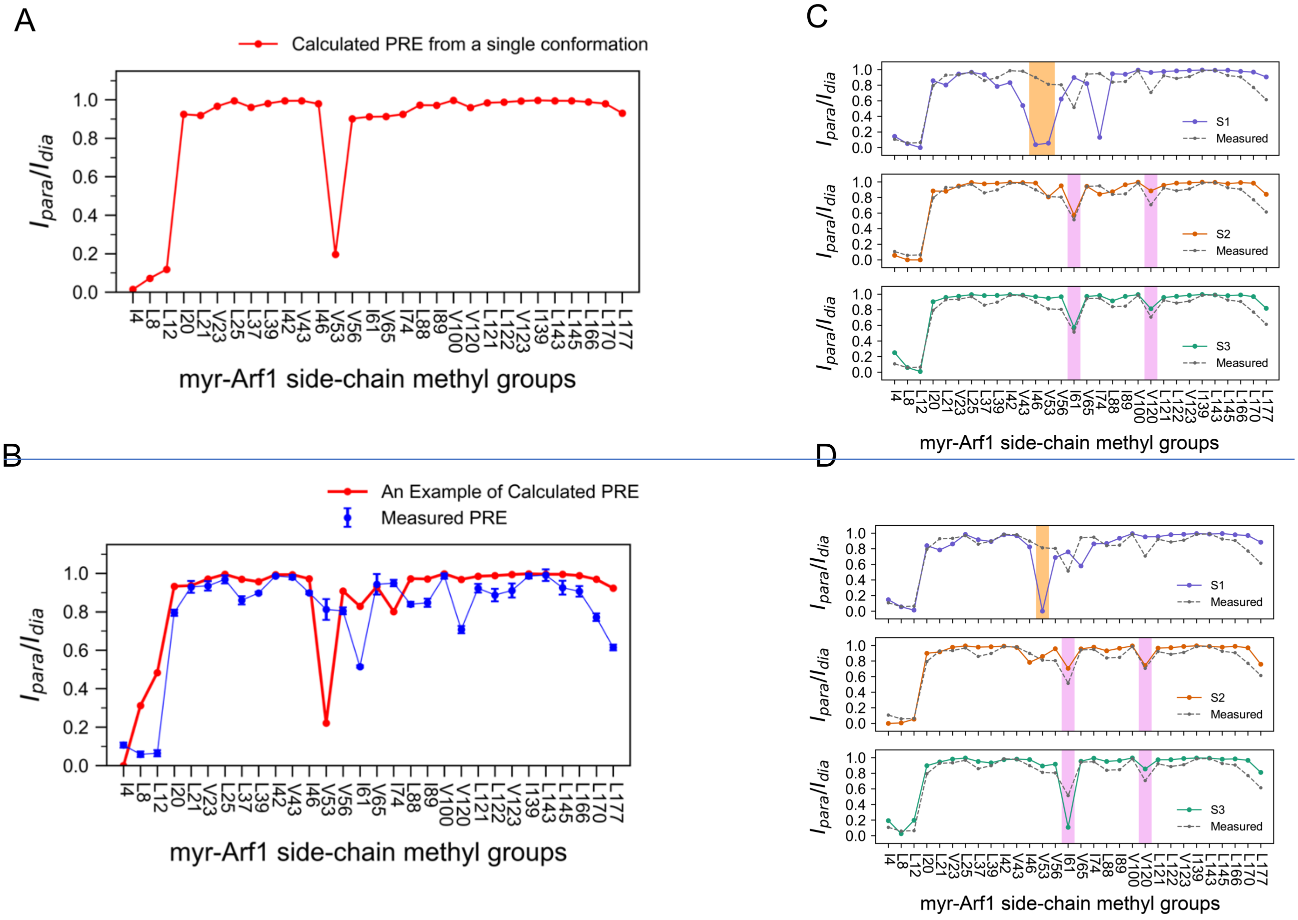

Supplementary Figure 12. Back calculated PRE versus reporter residue number. (A) a single conformation of the G-domain of myr-Arf1 bound to a POPC:PI(4,5)P_2 nanodisc is used to compute the back calculated PRE curve. (B) a comparison of the single conformation back calculation (red) with the observed PRE profile (blue). No single conformation yields a back-calculated PRE response curve that matches the measured PRE. Hence, an ensemble analysis selects conformations contributing to the observed PRE profile, as described below. (C,D) Plots of the average PRE for each of the S1, S2, S3 states compared to the experimentally observed PRE for the POPC:PI(4,5)P_2 and POPC:POPS:PI(4,5)P_2 nanodiscs, respectively. Source data are provided as a Source Data File.

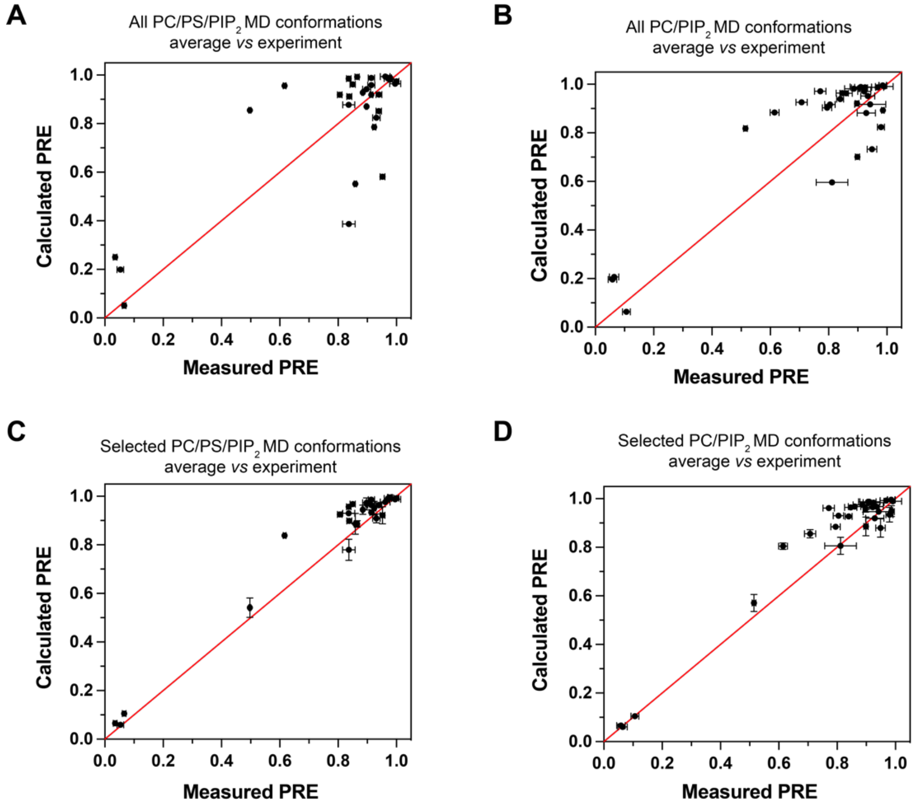

Supplementary Figure 13. Autocorrelation of calculated and measured PRE for different analyses using the MD-generated conformations. (A,B) The average of computed PRE for ***all*** MD conformations versus the measured PRE for (A) POPC/POPS/PI(4,5)P_2 and (B) POPC/PI(4,5)P_2 membranes. (C,D) The average of the best-fit ensemble MD conformations versus the measured PRE for (C) POPC/POPS/PI(4,5)P_2 and (D) POPC/PI(4,5)P_2 membranes. Error bars correspond to spectral signal-to-noise error in the x-axis (Measured PRE) and to +/- s in the y-axis (Calculated PRE). Source data are provided as a Source Data File.

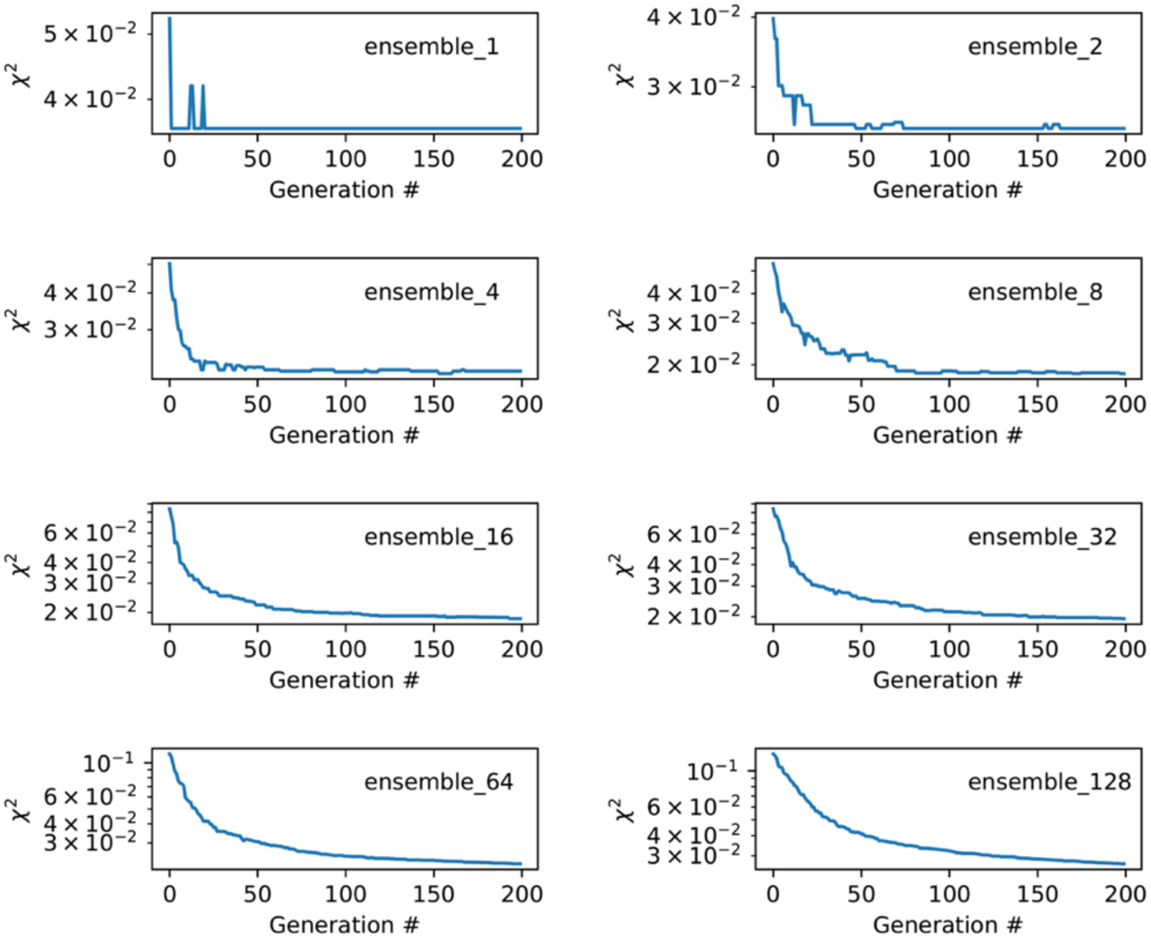

Supplementary Figure 14. The fitness score (χ^2) as a function of generation number with ensemble sizes of 1, 2, 4, 8, 16, 32, 64, 128. The lowest scores were reached after 200 generations for MD conformations. Source data are provided as a Source Data File.

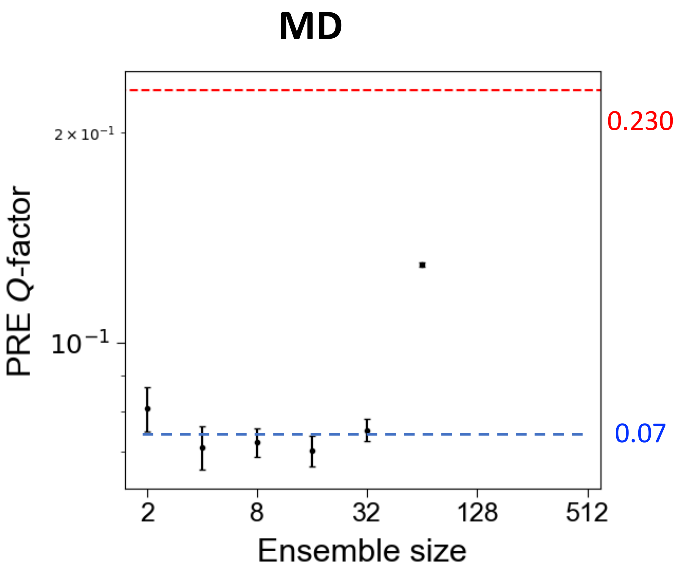

Supplementary Figure 15. Evaluation of PRE *Q*-factor [4-6] as a function of ensemble size and conformational database. The PRE Q-factor is used as a measure of performance, and is plotted as a function of ensemble size. The red dashed line is the average PRE *Q*-factor using all available structures in the respective databases, and the blue dashed line represents the average of PRE Q-factor for the selected ensemble members from each database. Error bars represent +/- σ. Source data are provided as a Source Data File.

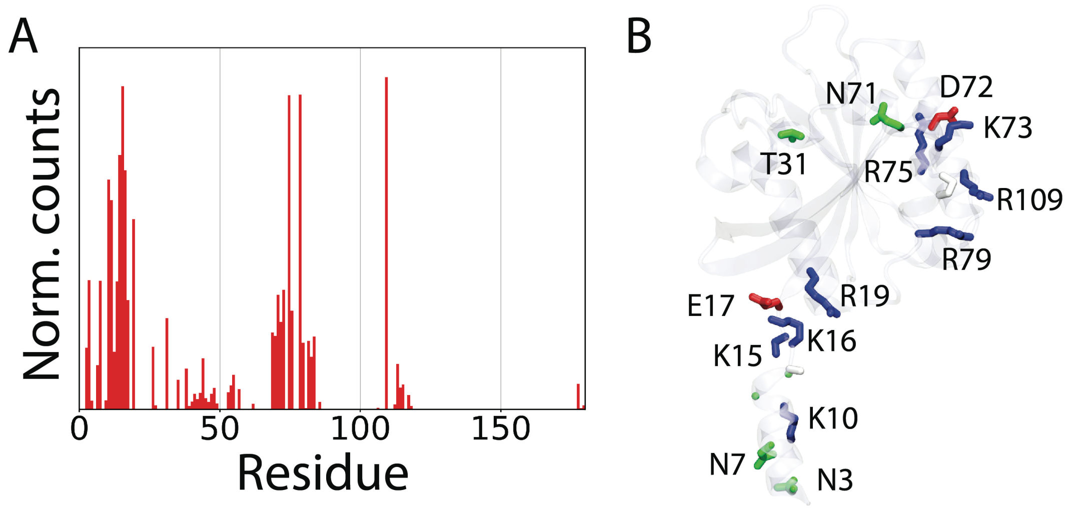

Supplementary Figure 16. Lipid-protein interactions in membrane-bound myr-Arf1. (A) A representative histogram of the protein residues in contact with the phosphate groups of PI(4,5)P_2 (P, P4, and P5). (B) A snapshot of membrane-bound myr-Arf1 highlighting the residues in contact with PI(4,5)P_2 is shown. All the lipid-protein contacts were analyzed using a representative 1000-ns full-membrane simulation. The residues are colored according to the residue-type: basic in blue, acidic in red, and polar in green. Source data are provided as a Source Data File.

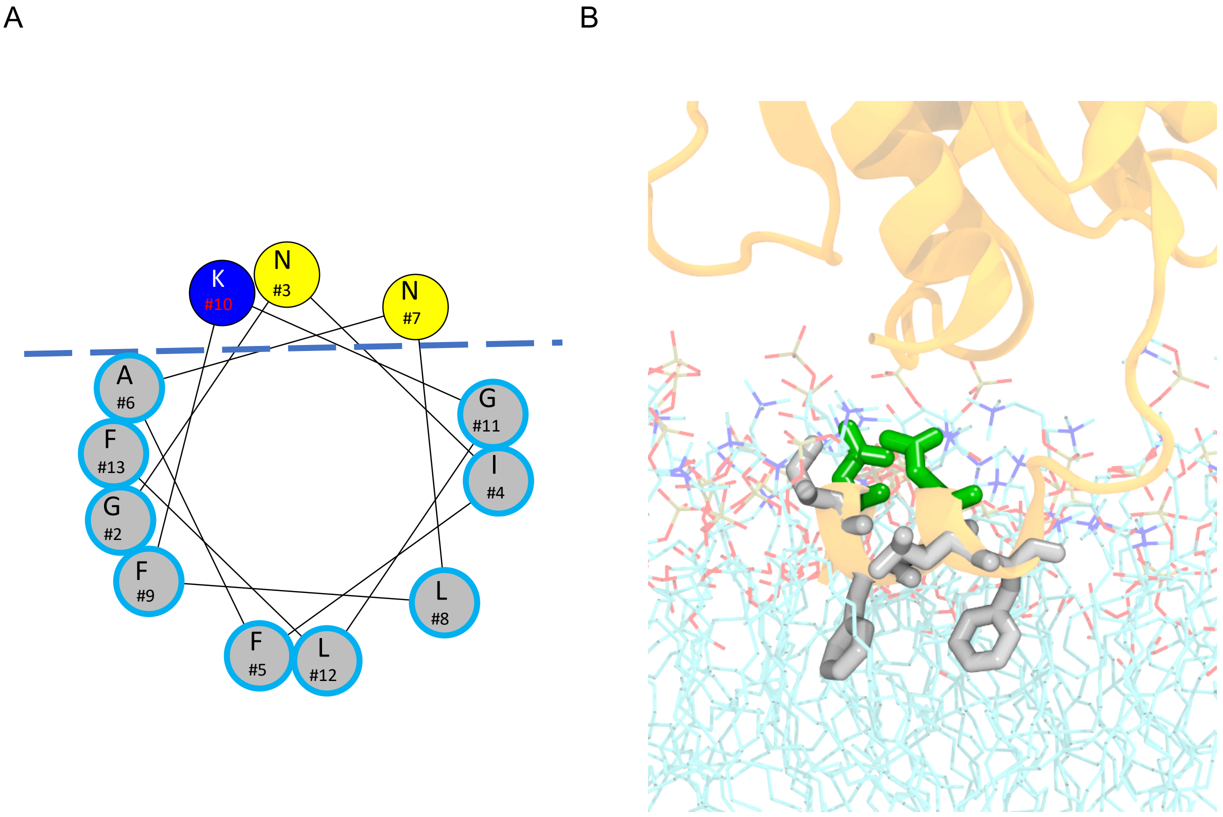

Supplementary Figure 17. The N-terminal helix of Arf1 is seen to bury in the membrane. (A) a helical wheel (generated with the program helixvis [https://pypi.org/project/helixvis/]) representation of the N-terminal helix position in the simulations, indicates that the hydrophobic and aromatic residues insert into the membrane below the phosphorus plane (dashed line) while the charged and hydrophilic residues remain above the plane, while. (B) Representative snapshot from the MD simulations that illustrates the residue positions shown in (A), with the solvent-exposed residue K10 (blue in A) and membrane-inserted residues F5, L12 and L8 (cyan in A).

Supplementary Table 1: Selected fit parameters and derived quantities for the NR measurements of 13.3 μM solution concentration activated myr-Arf1 added to POPC:POPS, POPC:PI(4,5)P_2 or POPC:POPS:PI(4,5)P_2 stBLMs. Molar ratios of the lipids constituting the stBLMs are given in the table header. The statistical analyses of the fit of the reflectivity were carried out with the reduced χ^2 method using composition-space modeling and the Monte-Carlo Markov chain global optimizer DREAM as implemented in the Refl1D software package.[2] Median fit parameter values and 68% confidence limits are reported. The protein is modeled as a free-form spline component volume occupancy (CVO) distribution. Roughness is given as a root mean square deviation from an ideally smooth interface. The POPC:POPS:PI(4,5)P_2 dataset has a higher substrate roughness that causes comparatively broader CVO profiles, as shown in Figure 2.

Parameter	POPC : POPS<br>85:15	POPC : PI(4,5)P_2<br>95:5	POPC : POPS : PI(4,5)P_2<br>87 : 10 : 3
Substrate			
Substrate roughness / Å	4.0 ± 0.6	3.5 ± 0.4	8 ± 1
SiOx thickness / Å	20 ± 10	30 ± 10	20 ± 10
SiOx nSLD / 10^{-6} Å^{-2}	3.36 ± 0.05	3.37 ± 0.02	3.1 ± 0.2
Cr thickness / Å	21 ± 9	20 ± 10	40 ± 10
Cr nSLD / 10^{-6} Å^{-2}	3.3 ± 0.1	3.34 ± 0.05	3.4 ± 0.2
Au thickness / Å	179 ± 2	137 ± 1	156 ± 5
Au nSLD / 10^{-6} Å^{-2}	4.46 ± 0.01	4.52 ± 0.01	4.34 ± 0.01
Bilayer			
Bilayer roughness / Å	4.0 ± 0.4	6.1 ± 0.2	8 ± 1
Thickness sub-membrane space / Å	8.4 ± 0.4	8.6 ± 0.2	9.5 ± 0.8
Substrate-proximal hydrocarbon thickness / Å	16.8 ± 0.7<br>Change after Arf1 addition: 0.0 ± 0.1	19.2 ± 0.5<br>Change after Arf1 addition: -0.1 ± 0.1	16.0 ± 0.9<br>Change after Arf1 addition: -0.9 ± 0.2
Substrate-distal hydrocarbon thickness	12.5 ± 0.8<br>Change after Arf1 addition: 0.0 ± 0.1	14.3 ± 0.5<br>Change after Arf1 addition: -0.1 ± 0.1	15.8 ± 1.0<br>Change after Arf1 addition: -0.9 ± 0.2
Bilayer completeness / %	98 ± 1<br>After Arf1 addition: 99 ± 1	100 ± 1<br>After Arf1 addition: 100 ± 1	98 ± 1<br>After Arf1 addition: 99 ± 1
Protein			
Volume surface density / Å^3/Å^2	3.7 ± 0.6	8.2 ± 0.5	11.7 ± 0.5
Fraction of protein outside of bilayer / %	87 ± 9	96 ± 4	91 ± 3
General			
Fit quality, χ^2	1.1	2.9	1.9

Supplementary Table 2: Simulation design and simulations performed on Arf1

Simulation type	Membrane Composition	# Replicas	Time (ns)/replica
HMMM*	POPC-POPS-PI(4,5)P_2 (75:20:5)	9	100
HMMM*	POPC-PI(4,5)P_2 (95:5)	9	100
HMMM*	POPC	9	100
Full Membrane**	POPC-POPS-PI(4,5)P_2 (75:20:5)	9	1000
Full Membrane**	POPC-PI(4,5)P_2 (95:5)	9	1000
Full Membrane**	POPC	9	1000

* Spontaneous membrane-binding simulations using HMMM membranes.

**Full Membrane simulations starting from membrane-bound replicas captured during HMMM membrane-binding simulations.

Supplementary Methods

High yield expression of isotopically labeled myr-Arf1

Reducing the proton background via perdeuteration is essential for solution-state NMR studies to limit relaxation and proton spin diffusion effects, which result in the subsequent loss of signal for complexes of large molecular weight, such as myr-Arf1∙GTP bound to a membrane-mimic nanodisc (~140 kDa). Uniform ^{15}N-labeling provides information for all residues along the protein backbone. Specific ^{13}C-labeling of the side-chain methyl groups of isoleucine (I), leucine (L), and valine (V) residues can be combined with [$^{1}H-^{13}C$]-methyl-Heteronuclear Multiple Quantum Correlation (HMQC) NMR to observe the resonances of these methyl groups in very large biomolecular particles, even for complexes >300 kDa[7]. In a previous study, we demonstrated efficient production of N-myristoylated, U-^{15}N and ^{13}C-methyl labeled myr-Arf1 using a codon-optimized, dual plasmid expression approach[2]. However, expression in D_2O-based minimal media, supplemented by deuterated glucose as a carbon source (for perdeuteration)[8], yielded poor protein expression. We postulated that combined exposure to multiple antibiotics, minimal media, and D_2O collectively increase the stress on the bacterium and negatively impact expression. Inspired by the work of Glück et al., in which high yield expression and efficient HIV1-Nef myristoylation were achieved with a single vector system, we used the single vector system pETDuet-1 (Novagen) for cloning and co-expression of Arf1 and human N-myristoyl transferase (hNMT-1) genes. Typical yield in the single vector system was 7-8 mg of [$^{2}H,^{15}N$]; Ile δ1-[$^{13}CH_3$]; Leu, Val [$^{13}CH_3,^{12}CD_3$] labeled myr-Arf1 per liter of culture. An approximately two-fold improvement in sensitivity and resolution of a $^{1}H-^{13}C$ HMQC experiment recorded on a 140 kDa complex composed of two perdeuterated myr-Arf1∙GTP bound to a nanodisc (one per side) is shown in Supplementary Figure 3.

NMR PRE data analyses

Analysis of G-domain conformations via back-calculation of PRE based on atomic coordinates from MD simulation trajectories

Paramagnetic Relaxation Enhancement (PRE) effects can provide long-range distance information and have been widely used as restraints for constructing a conformational ensemble to describe a dynamic system [9]. The PRE for an observed proton (e.g. a methyl proton in the myr-Arf1 protein) resulting from a paramagnetic spin label (located in the ND membrane) can be readily calculated for a single conformation based on the distance between the paramagnetic center and the observed proton. For multi-state systems, such as a dilute doxyl-lipid in a ND, a few assumptions are required to enable computing the PRE effect from the doxyl group to a surface-bound protein molecule. First, we assumed that lipid lateral diffusion is fast enough such that the doxyl tagged lipid can occupy all *N* positions in the membrane leaflet of the ND [10,11]. Second, we require a set of conformational states for the protein relative to the membrane.

Atomic coordinates of lipids and of the Arf1 protein were extracted from the MD trajectories (1 frame every 6 ns for each of the nine 1 µs long simulations for a total of ~ 1500 frames) and used to back calculate PRE rates. The membrane was treated as the closest 100 lipid molecules (50 lipids per leaflet). The coordinates of the 5th acyl chain carbon of the POPC molecule were chosen as the depth of the doxyl tag free electron. For each of the ~ 1500 frames, the effective PRE relaxation rate ($\mathit{\Gamma}_2^{cal}$) (Supplementary equation 1) of a protein methyl proton was then calculated as the average of the PRE rate $\mathit{\Gamma}_{2,i}$ (Supplementary equation 2) computed for each of the N possible 5-doxyl-PC lipid positions in the bilayer:

$$\mathit{\Gamma}_2^{cal} = \frac{1}{N}\sum_{i=1}^{N}\mathit{\Gamma}_{2,i} \qquad (1)$$

with $\mathit{\Gamma}_{2,i}$ equal to:

$$\mathit{\Gamma}_{2,i} = \kappa\left(4\tau_c + \frac{3\tau_c}{1+\omega_H^2\tau_c^2}\right)r^{-6} \qquad (2)$$

with κ equal to 1.23 × 10^{-32} cm^6s^{-2} for the proton spin as reported previously [12], r the distance between the free electron and methyl group protons of I, L or V residues in a single frame, τ_c the rotational correlation time of the electron-nuclear interaction, which was approximated using Supplementary equation 3

$$\frac{1}{\tau_c} = \frac{1}{\tau_R} + \frac{1}{\tau_S} \qquad (3)$$

where τ_R is protein rotational correlation time and τ_S is the electronic longitudinal relaxation time. We used a τ_R of 34 ns (measured from a TRACT experiment on the G-domain of myr-Arf1) and a value of 100 ns [12] for τ_S. Then, the Γ_2^{cal} were converted to the intensity ratios of the paramagnetic to diamagnetic peaks (I_{para}/I_{dia}) using Supplementary equation 4:

$$\frac{I_{para}}{I_{dia}} = \frac{R_2 \times \exp(-\Gamma_2^{cal} \times t)}{R_2 + \Gamma_2^{cal}} \qquad (4)$$

where t is the total evolution time of 6.89 ms in the HQMC pulse sequence, R_2 is the intrinsic transverse relaxation rate, which was estimated from the half-height line width of peaks in the diamagnetic spectra. An example of back calculated PRE values plotted versus residue number is shown in Supplementary Figure S12. Calculation was then repeated for each frame and back calculated PRE values were then utilized in an ensemble analysis to select the ensemble of conformations that best match the experimental data.

Selection of optimal ensembles based on experimental PRE measurements.

A genetic algorithm was used, starting from 128 randomly selected ensembles, each containing *N* structures (selected from either the 1465 or 1643 structures from MD for POPC/PI(4,5)P_2 and POPC/POPS/PI(4,5)P_2 membrane, respectively), which is called a *generation*. Based on those ensembles, a larger population of ensembles were generated by the *evolution* process, which includes three biologically inspired operators: *selection*, *mutation*, and

crossover [13]. The new ensembles were further individually evaluated by a fitness function (Supplementary equation 5) comparing the back calculated paramagnetic relaxation rates Γ_2^{cal} to the experimental Γ_2^{obs} measurements:

$$\chi^2 = \sum_i^k \left(\frac{\Gamma_2^{obs}(i) - \Gamma_2^{cal}(i)}{\Gamma_2^{obs}(i)} \times SF_i \right)^2 \qquad (5)$$

where $\Gamma_2^{cal}(i)$ is the ensemble-averaged PRE value for residue i; k is the total number of residues used in the calculation; *SF$_i$* is a scaling factor (a value of 2 was used for the residues included in the selection algorithm). The candidates then randomly split into groups for tournaments to provide the next *generation*. The winners of tournaments were kept as the *parents* for next *generation*. The number of tournaments successively went from 128, 64, 32, 16, and 2 to obtain the final winner. To ensure robust fitting [14], a total of 200 generations were performed (SI Figure S13).

To determine the optimal ensemble size (*N*) for selection, a series of ensemble sizes (2, 4, 8, 16, 32, and 64) were tested and evaluated by the average PRE *Q*-factor [4-6] (Supplementary equation 6):

$$Q = \frac{1}{N} \sqrt{\frac{\sum_i^n \{\Gamma_2^{obs}(i) - \Gamma_2^{cal}(i)\}^2}{\sum_i^n \Gamma_2^{obs}(i)^2}} \qquad (6)$$

To avoid the artifacts from the experimental error and the additional C-terminal 6-His Tag, the experimental PRE values greater than 1 and the C-terminal region (representing reporting residues 166, 170, 173, and 177) were not used as experimental restraints in the selection process.

The selection algorithm was optimized to ensure the best performance with the lowest χ^2 was achieved (SI Figure S13). To account for experimental errors, 100 sets of the selection

restraints/inputs were generated by a *monte-carlo* method, randomly inserting 15% error based on the observed experimental errors (main text Figure 5C and 5G, red curves). The performance based on ensemble size of 16 was chosen for further analysis. The resulting 100 ensembles, each comprising of 16 structures without a duplication, achieves excellent agreement with the experimental data with the average PRE *Q*-factor of 0.05 (Figure 5E,I and SI Figure S14). This protocol is related to the repetitive computation of NMR structures from NOE distance restraints and yields a set of 100 x 16 conformations that satisfy the experimental restraints. Within the combined set, numerous conformations are repetitively selected, and the distribution is shown in Figures 5D,H.

Molecular dynamics simulation

Analysis of Simulation results

All analyses were performed in VMD (55). The membrane-binding configuration and depth of Arf1 insertion into the lipid bilayer were monitored by calculating the ensemble-averaged *z* positions of all C_α atoms with respect to the cis-leaflet phosphate plane. The relative location of the G domain (specified as residues > 18) of Arf1 with respect to the phosphorous plane was gauged by calculating its COM. We have also quantified specific lipid-protein interactions between the lipid headgroups and Arf1 residues. Based on our previous studies [15], a 3.5-Å heavy atom cutoff was used to define specific lipid-protein contacts. Furthermore, to describe the lipid-dependent orientational dynamics of Arf1, we computed the rotation matrices that transform each frame of full tail simulations to a reference position that can minimize the root mean square displacement (RMSD) between the two orientations. The rotation matrices were calculated for the backbone heavy atoms of the relatively stable G domain (residues > 18). The rotation matrix was obtained by calculating the Euler angles and described by $R(\alpha, \beta, \gamma)$ (Supplementary equation 7):

$$R(\alpha,\beta,\gamma) = \begin{pmatrix} \cos\alpha\cos\beta\cos\gamma - \sin\alpha\sin\gamma & -\cos\alpha\cos\beta\sin\gamma - \sin\alpha\cos\gamma & \cos\alpha\sin\beta \\ \sin\alpha\cos\beta\cos\gamma + \cos\alpha\sin\gamma & -\sin\alpha\cos\beta\sin\gamma + \cos\alpha\cos\gamma & \sin\alpha\sin\beta \\ -\sin\beta\cos\gamma & \sin\beta\sin\gamma & \cos\beta \end{pmatrix} \quad (7)$$

All the rotation matrices were calculated using the 'measure fit' command in VMD. We chose a reference structure wherein helix 5 of Arf1 was perpendicular to the membrane so that α has a relatively uniform distribution for the most probable values of β, γ [16]. The two Euler angles (β, γ) were used to characterize the orientational dynamics of the protein.

Supplementary References

1 Webb, B. & Sali, A. Comparative Protein Structure Modeling Using MODELLER. *Curr Protoc Bioinformatics* **54**, 5 6 1-5 6 37 (2016). https://doi.org/10.1002/cpbi.3

2 Li, Y. *et al.* Functional Expression and Characterization of Human Myristoylated-Arf1 in Nanodisc Membrane Mimetics. *Biochemistry* **58**, 1423-1431 (2019). https://doi.org/10.1021/acs.biochem.8b01323

3 Kienzle, P. A. *et al. Refl1D Reflectometry Software*, <https://www.nist.gov/ncnr/reflectometry-software> (2017).

4 Iwahara, J., Anderson, D. E., Murphy, E. C. & Clore, G. M. EDTA-derivatized deoxythymidine as a tool for rapid determination of protein binding polarity to DNA by intermolecular paramagnetic relaxation enhancement. *J Am Chem Soc* **125**, 6634-6635 (2003).

5 Iwahara, J., Schwieters, C. D. & Clore, G. M. Ensemble approach for NMR structure refinement against (1)H paramagnetic relaxation enhancement data arising from a flexible paramagnetic group attached to a macromolecule. *J Am Chem Soc* **126**, 5879-5896 (2004).

6 Clore, G. M. & Iwahara, J. Theory, practice, and applications of paramagnetic relaxation enhancement for the characterization of transient low-population states of biological macromolecules and their complexes. *Chem Rev* **109**, 4108-4139 (2009).

7 Rosenzweig, R. & Kay, L. E. Bringing dynamic molecular machines into focus by methyl-TROSY NMR. *Annu Rev Biochem* **83**, 291-315 (2014). https://doi.org/10.1146/annurev-biochem-060713-035829

8 Li, J. & Byrd, R. A. A simple protocol for the production of highly deuterated proteins for biophysical studies. *J Biol Chem* **298**, 102253 (2022). https://doi.org/10.1016/j.jbc.2022.102253

9 Clore, G. M., Tang, C. & Iwahara, J. Elucidating transient macromolecular interactions using paramagnetic relaxation enhancement. *Curr Opin Struct Biol* **17**, 603-616 (2007).

10 Marcink, T. C. *et al.* MT1-MMP Binds Membranes by Opposite Tips of Its beta Propeller to Position It for Pericellular Proteolysis. *Structure* **27**, 281-292 e286 (2019). https://doi.org/10.1016/j.str.2018.10.008

11 Mazhab-Jafari, M. T. *et al.* Membrane-dependent modulation of the mTOR activator Rheb: NMR observations of a GTPase tethered to a lipid-bilayer nanodisc. *J Am Chem Soc* **135**, 3367-3370 (2013). https://doi.org/10.1021/ja312508w

12 Battiste, J. L. & Wagner, G. Utilization of site-directed spin labeling and high-resolution heteronuclear nuclear magnetic resonance for global fold determination of large proteins with limited nuclear overhauser effect data. *Biochemistry* **39**, 5355-5365 (2000).

13 Fraser, A. Simulation of Genetic Systems by Automatic Digital Computers I. Introduction. *Australian Journal of Biological Sciences* **10**, 484-491 (1957). https://doi.org/https://doi.org/10.1071/BI9570484

14 Salmon, L. *et al.* NMR characterization of long-range order in intrinsically disordered proteins. *J Am Chem Soc* **132**, 8407-8418 (2010). https://doi.org/10.1021/ja101645g

15 Pant, S. & Tajkhorshid, E. Microscopic Characterization of GRP1 PH Domain Interaction with Anionic Membranes. *J Comput Chem* **41**, 489-499 (2020). https://doi.org/10.1002/jcc.26109

16 Ngo, V. A., Sarkar, S., Neale, C. & Garcia, A. E. How Anionic Lipids Affect Spatiotemporal Properties of KRAS4B on Model Membranes. *J Phys Chem B* **124**, 5434-5453 (2020). https://doi.org/10.1021/acs.jpcb.0c02642

2B. Raw image file for Supplementary Figure

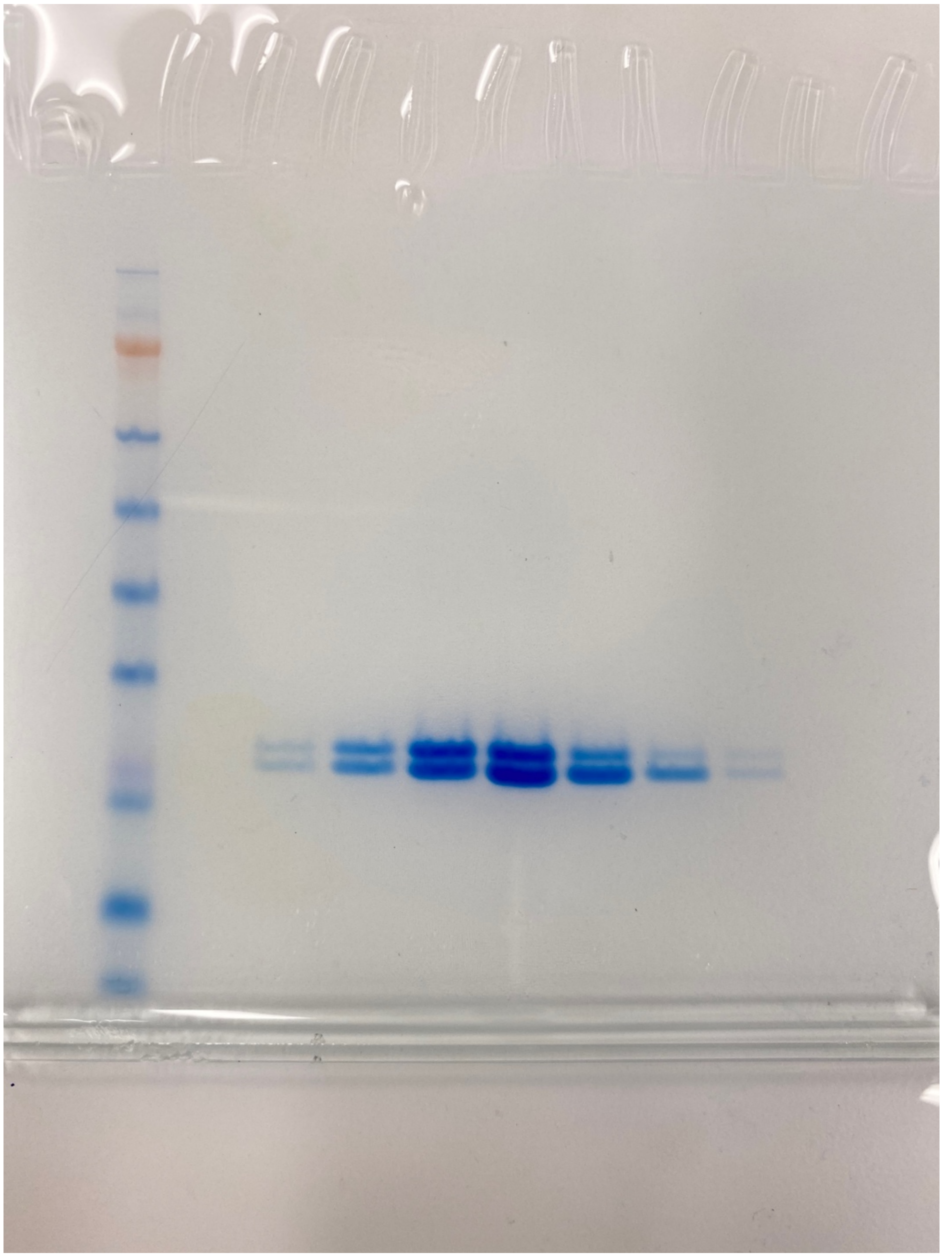

Supplement: Supplementary file 1 — Supplementary Information [file 41467_2023_43008_MOESM1_ESM.pdf]
