# Supplementary material for: Myr-Arf1 conformational flexibility at the membrane surface sheds light on the interactions with ArfGAP ASAP1
Source: Nat Commun. 2023 Nov 21;14:7570. doi: 10.1038/s41467-023-43008-5 (PMC10663523; doi:10.1038/s41467-023-43008-5)
Supplement: Supplementary file 3 — Reporting Summary [file 41467_2023_43008_MOESM3_ESM.pdf]

nature portfolio

Corresponding author(s): R. Andrew Byrd

Last updated by author(s): Oct 24, 2023

Reporting Summary

Nature Portfolio wishes to improve the reproducibility of the work that we publish. This form provides structure for consistency and transparency in reporting. For further information on Nature Portfolio policies, see our Editorial Policies and the Editorial Policy Checklist.

Please do not complete any field with "not applicable" or n/a. Refer to the help text for what text to use if an item is not relevant to your study.
For final submission: please carefully check your responses for accuracy; you will not be able to make changes later.

Statistics

For all statistical analyses, confirm that the following items are present in the figure legend, table legend, main text, or Methods section.

n/a	Confirmed	
☐	☒	The exact sample size (*n*) for each experimental group/condition, given as a discrete number and unit of measurement
☐	☒	A statement on whether measurements were taken from distinct samples or whether the same sample was measured repeatedly
☐	☒	The statistical test(s) used AND whether they are one- or two-sided<br>*Only common tests should be described solely by name; describe more complex techniques in the Methods section.*
☒	☐	A description of all covariates tested
☒	☐	A description of any assumptions or corrections, such as tests of normality and adjustment for multiple comparisons
☐	☒	A full description of the statistical parameters including central tendency (e.g. means) or other basic estimates (e.g. regression coefficient) AND variation (e.g. standard deviation) or associated estimates of uncertainty (e.g. confidence intervals)
☐	☒	For null hypothesis testing, the test statistic (e.g. *F*, *t*, *r*) with confidence intervals, effect sizes, degrees of freedom and *P* value noted<br>*Give P values as exact values whenever suitable.*
☒	☐	For Bayesian analysis, information on the choice of priors and Markov chain Monte Carlo settings
☒	☐	For hierarchical and complex designs, identification of the appropriate level for tests and full reporting of outcomes
☒	☐	Estimates of effect sizes (e.g. Cohen's *d*, Pearson's *r*), indicating how they were calculated

Our web collection on statistics for biologists contains articles on many of the points above.

Software and code

Policy information about availability of computer code

Data collection	NMR data was collected with Bruker TopSpin 3.6.5 software supplied with NMR instrumentation. NR data was collected with software specific to the neutron reactor facility (NIST Center for Neutron Research, Maryland, U.S. and ISIS Neutron and Muon Source, Didcot, UK).
Data analysis	NMR data processing (NMRPipe) and analysis (NMRFAM-Sparky, nightshift) software was used and cited in the manuscript. Custom software written for NMR analyses is available via Zenodo as stated in the Code availability statement. Molecular dynamics using HMMM in combination with NAMD2 and CHARMM36 is described in the Code availability statement. Molecular images were created using Chimera 1.17.3 or earlier.

For manuscripts utilizing custom algorithms or software that are central to the research but not yet described in published literature, software must be made available to editors and reviewers. We strongly encourage code deposition in a community repository (e.g. GitHub). See the Nature Portfolio guidelines for submitting code & software for further information.

Data

Policy information about availability of data

All manuscripts must include a data availability statement. This statement should provide the following information, where applicable:

- Accession codes, unique identifiers, or web links for publicly available datasets
- A description of any restrictions on data availability
- For clinical datasets or third party data, please ensure that the statement adheres to our policy

The datasets generated and analyzed in the current study and presented as the main and supplementary figures are available in the Figshare repository with the identifier https://doi.org/10.6084/m9.figshare.23846991. All other data that support the findings of this study will be available from the corresponding authors upon reasonable request. Data is cited from public databases (Protein Data Bank and UniProt) and the citations include appropriate hyperlinks to the content used.

Human research participants

Policy information about studies involving human research participants and Sex and Gender in Research.

Reporting on sex and gender	N/A
Population characteristics	N/A
Recruitment	N/A
Ethics oversight	N/A

Note that full information on the approval of the study protocol must also be provided in the manuscript.

Field-specific reporting

Please select the one below that is the best fit for your research. If you are not sure, read the appropriate sections before making your selection.

☒ Life sciences ☐ Behavioural & social sciences ☐ Ecological, evolutionary & environmental sciences

For a reference copy of the document with all sections, see nature.com/documents/nr-reporting-summary-flat.pdf

Life sciences study design

All studies must disclose on these points even when the disclosure is negative.

Sample size	No statistical methods were used to predetermine sample size. For all data sets provided in this work, we chose replicate size according to standard methods provided in the literature and to our own preliminary experiments so as to provide sufficient power for statistical comparison (where applicable).
Data exclusions	No data were excluded from the analyses. All data mentioned in the manuscript were used in the analyses.
Replication	All attempts at replication were successful. The numbers of experiments are described in the Methods section and figure legends, where applicable.
Randomization	Randomization method was not utilized. This was not a clinical trial (i.e., no animals or humans were involved) and different bacterial strains were not being compared to each other.
Blinding	Blinding is not relevant for this study because no group allocation was performed

Reporting for specific materials, systems and methods

We require information from authors about some types of materials, experimental systems and methods used in many studies. Here, indicate whether each material, system or method listed is relevant to your study. If you are not sure if a list item applies to your research, read the appropriate section before selecting a response.

Materials & experimental systems

n/a	Involved in the study
☒	☐ Antibodies
☒	☐ Eukaryotic cell lines
☒	☐ Palaeontology and archaeology
☒	☐ Animals and other organisms
☒	☐ Clinical data
☒	☐ Dual use research of concern

Methods

n/a	Involved in the study
☒	☐ ChIP-seq
☒	☐ Flow cytometry
☒	☐ MRI-based neuroimaging

This checklist template is licensed under a Creative Commons Attribution 4.0 International License, which permits use, sharing, adaptation, distribution and reproduction in any medium or format, as long as you give appropriate credit to the original author(s) and the source, provide a link to the Creative Commons license, and indicate if changes were made. The images or other third party material in this article are included in the article's Creative Commons license, unless indicated otherwise in a credit line to the material. If material is not included in the article's Creative Commons license and your intended use is not permitted by statutory regulation or exceeds the permitted use, you will need to obtain permission directly from the copyright holder. To view a copy of this license, visit http://creativecommons.org/licenses/by/4.0/
